# Pigmented Villonodular Synovitis of the Glenohumeral Joint and Biceps Tendon Sheath

**DOI:** 10.7759/cureus.14529

**Published:** 2021-04-17

**Authors:** Eli T Sayegh, Richard M Wilk

**Affiliations:** 1 Orthopaedic Surgery, Brigham and Women's Hospital, Harvard Medical School, Boston, USA

**Keywords:** pigmented villonodular synovitis, pvns, glenohumeral joint, shoulder, biceps tendon sheath, arthroscopy

## Abstract

A 34-year-old woman presented with paroxysmal, insidious shoulder pain with effusion. MRI demonstrated a permeative, intermediate-signal lesion on T1 and T2 sequences involving the glenohumeral joint and biceps tendon sheath. The patient was treated with arthroscopic synovectomy, debridement, and subpectoral biceps tenodesis, with histopathology demonstrating pigmented villonodular synovitis (PVNS).

PVNS is an extremely rare lesion of the glenohumeral joint and surrounding extra-articular structures. Awareness of this condition is paramount for timely diagnosis and intervention before joint destruction occurs. Arthroscopic treatment with meticulous attention to surgical technique is a feasible treatment strategy in the absence of end-stage chondral damage.

## Introduction

Pigmented villonodular synovitis (PVNS) is a proliferative, typically monoarticular condition of the synovium occurring in 1.8 per million people [1]. This condition most commonly arises in the fourth to sixth decades of life and has no clear gender predilection [2]. Although both neoplastic and inflammatory etiologies have been proposed, its pathogenesis remains uncertain [3]. PVNS occurs in either localized or diffuse forms, of which the latter is more common, is more rapidly destructive, and carries a poorer prognosis [3]. The localized form causes a focal, pedunculated, nodular synovial lesion, whereas the diffuse form is characterized by global synovitis with a proclivity for extra-articular extension and higher risk of postoperative disease recurrence. PVNS is characterized by the insidious onset of atraumatic and often intermittent pain, recurrent effusion, and painful shoulder motion, which frequently result in diagnostic and treatment delay. In a series of diffuse-type knee PVNS, only 17% of patients were appropriately diagnosed prior to referral [4]. Radiographs have low diagnostic yield but occasionally demonstrate periarticular erosions or early degenerative changes. MRI is the mainstay of the diagnostic workup and demonstrates a periarticular or synovial nodular mass, so-called “blooming artifact,” [5] reciprocal periarticular bony lesions, and/or low signal on T1 and T2 sequences (“dark on dark”) signifying hemosiderin deposition. Histopathological findings of hemosiderin deposition, lipid-laden histiocytes, multinucleated giant cells, and stromal and fibroblast cell proliferation confirm the diagnosis [6].

While PVNS has been described in numerous joints, chiefly the knee, reports of shoulder involvement are extremely rare. Previous case reports have linked shoulder PVNS with massive, often irreparable rotator cuff tears [7-15] and chondral lesions [7,10,16,17]. Numerous treatment modalities have been proposed, including open or arthroscopic synovectomy and debridement [7,8,10,12,18] versus arthroplasty [16,19,20], with or without the use of adjuvant radiation [8], depending on the severity and extent of intra-articular disease, presence of extra-articular disease, and surgeon experience. In this case report, we describe a case of insidious PVNS involving the glenohumeral joint and biceps tendon sheath that was treated with arthroscopic synovectomy, debridement, and subpectoral biceps tenodesis.

## Case presentation

A 34-year-old woman reported the onset of atraumatic, episodic shoulder pain initially beginning in February 2019 with spontaneous resolution. She presented to the emergency department with recurrent pain three months later and was referred to our orthopedic clinic. She described the pain as intermittent, associated with pressure-like discomfort, and occasionally radiating into the arm. She reported pain at nighttime and with overhead and behind-back activity. Physical examination of the shoulder demonstrated a full functional arc of motion except for discomfort reaching behind her back. She exhibited full strength with resisted supraspinatus, infraspinatus, and subscapularis testing. Radiographs demonstrated inferior humeral head subluxation, without other skeletal or articular abnormalities. Nonsteroidal anti-inflammatory drugs (NSAIDs) and physical therapy for strengthening were prescribed due to a suspicion of ligamentous laxity in the shoulder, though the patient opted to perform home exercises.

**Figure 1:**
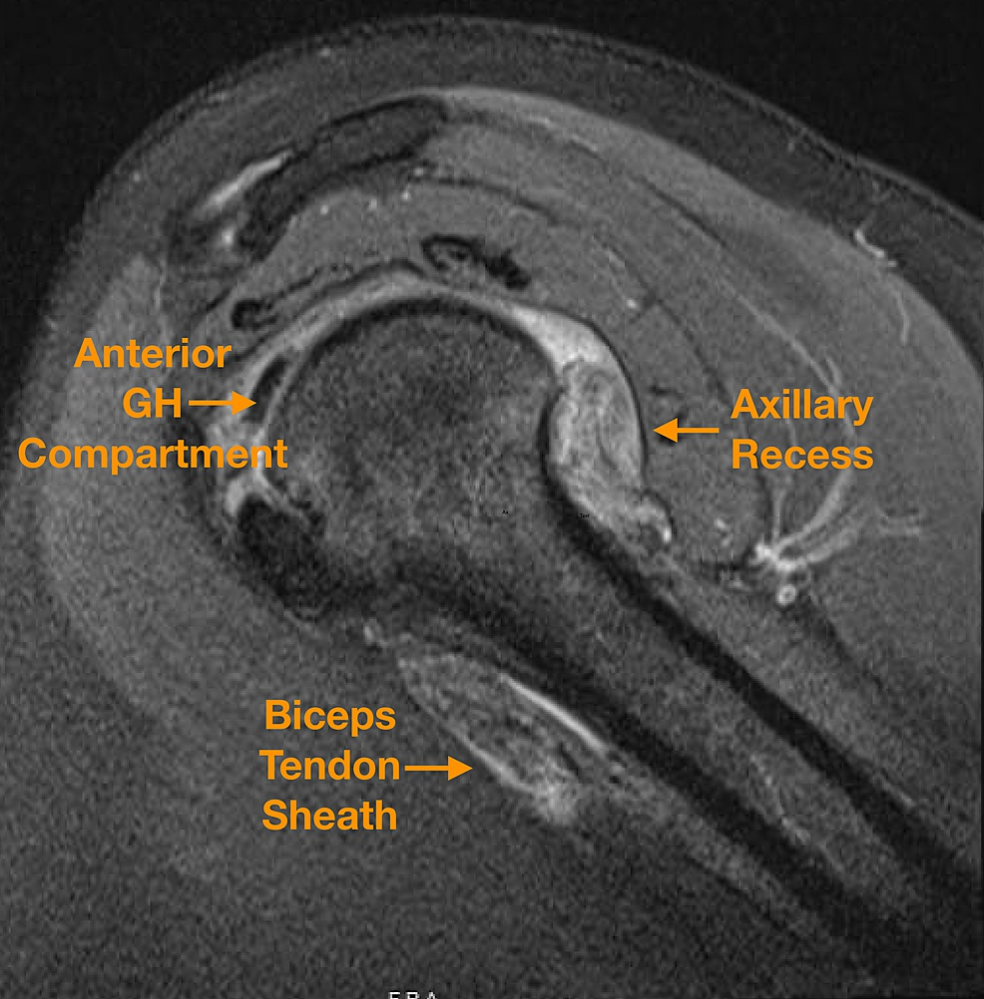
Sagittal proton-density fat-saturated MRI sequences demonstrating an intermediate-intensity, permeative, lobular lesion within the anterior glenohumeral (GH) compartment, axillary recess, and biceps tendon sheath.

The patient returned for evaluation 16 months later, when she presented again with persistent, activity-limiting pain rated at a severity of 9 of 10. Physical examination at that visit was notable for mild limitation of motion with forward flexion, abduction, external rotation, and internal rotation, as well as a positive impingement sign. In light of her protracted symptoms and worsening examination despite nonoperative treatment, an MRI was ordered, which demonstrated a heterogeneous, lobular, permeative intra-articular lesion with low-to-intermediate signal on both T1 and T2 sequences (Figure 1). Global glenohumeral involvement was noted including within the axillary recess, subscapularis recess, and anterior glenohumeral compartment deep to the subscapularis tendon, with extra-articular extension into the biceps tendon sheath. Because of the atypical MRI features, a screening laboratory panel for inflammatory or infectious disease was ordered, with anti-nuclear antibody, white blood cell count, erythrocyte sedimentation rate, and C-reactive protein all within normal limits.

**Figure 2:**
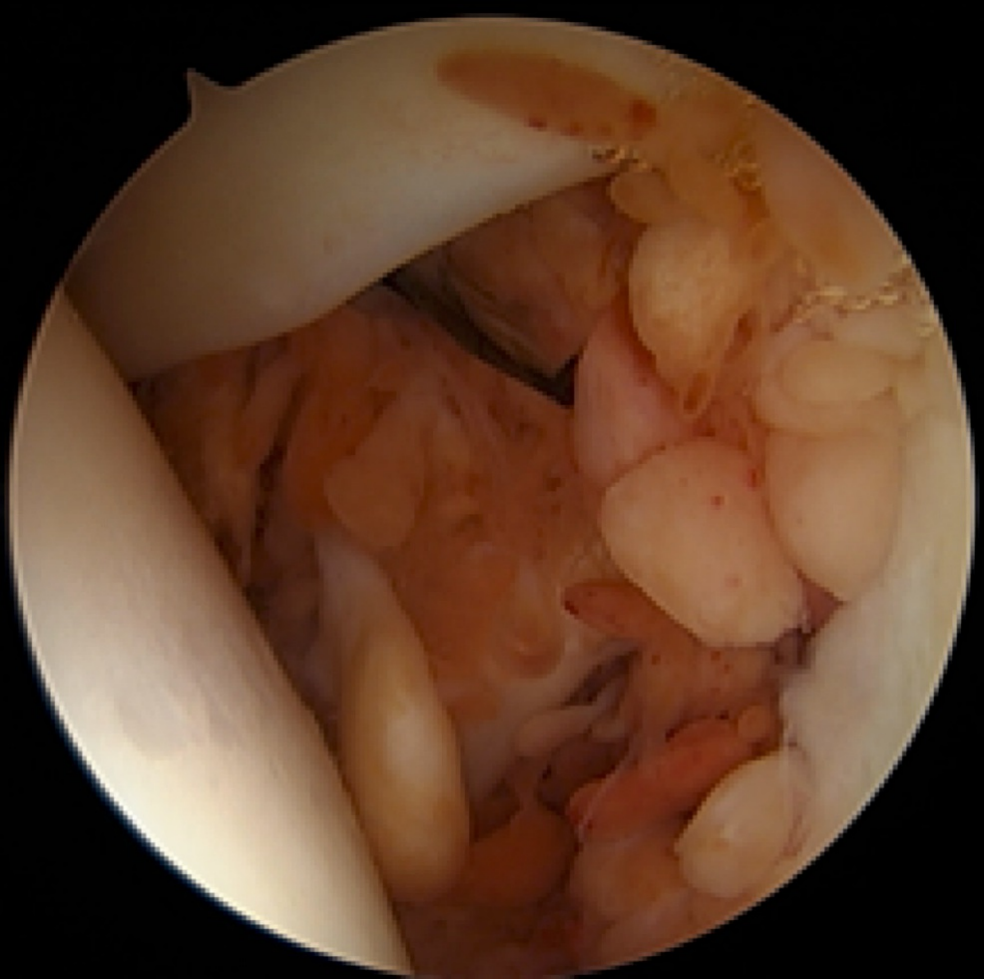
Arthroscopic image of proliferative synovitis with numerous yellow-brown to rust-colored papillary projections situated within the left glenohumeral joint. Humeral head (left), glenoid and labrum (right), subscapularis (center), and biceps tendon (top) are depicted.

**Figure 3:**
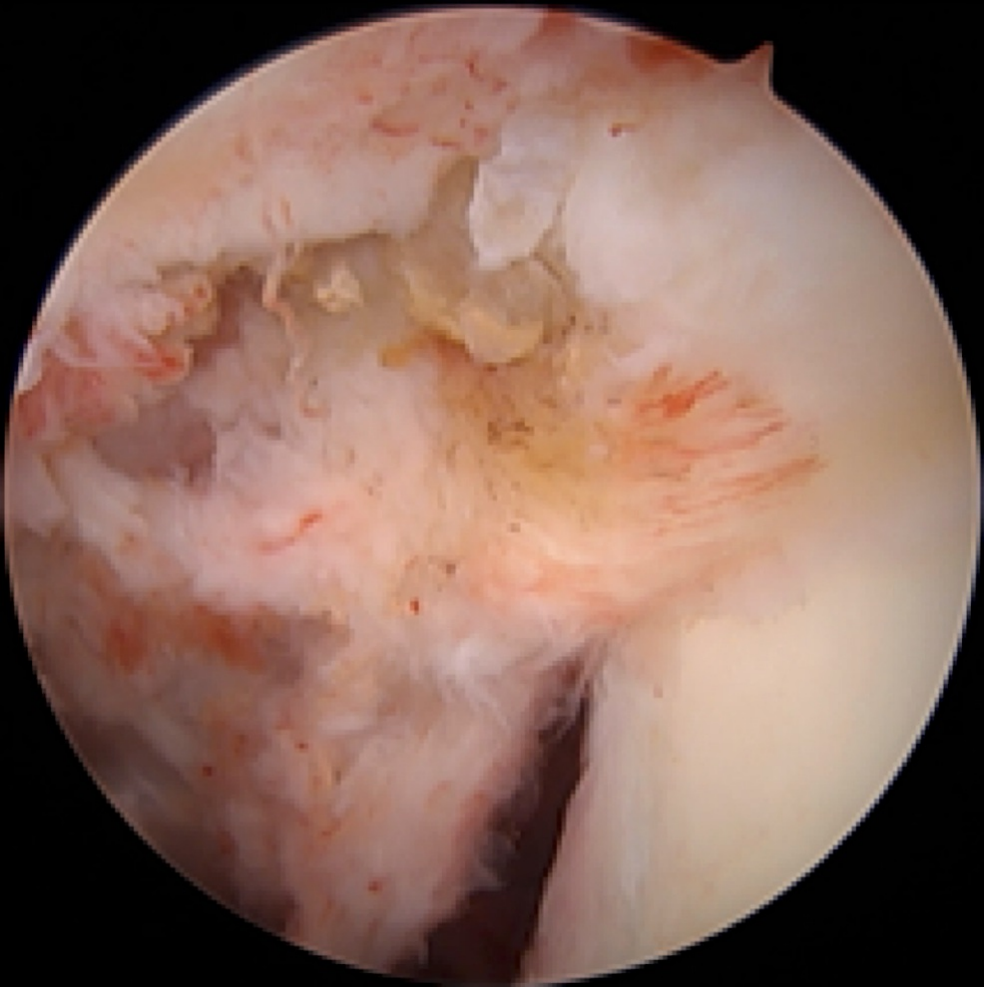
Arthroscopic image demonstrating disease removal following arthroscopic synovectomy and debridement of the left glenohumeral joint.

**Figure 4:**
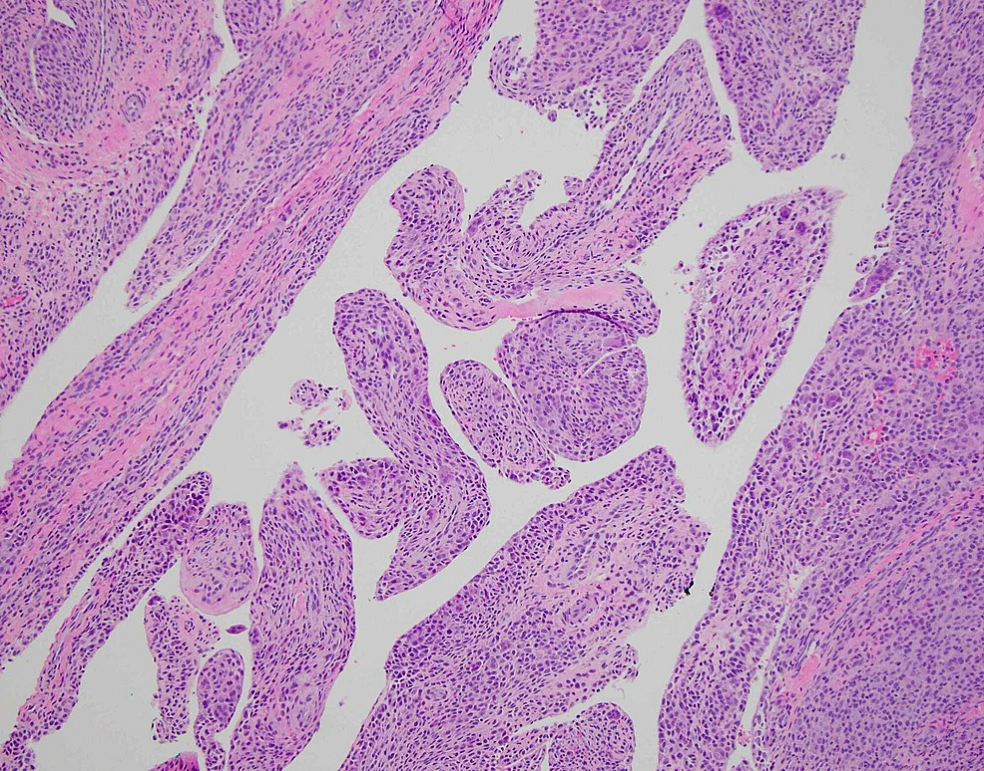
Low-power photomicrograph showing the villous architecture of proliferation, with villi composed of many mononuclear cells and few giant (multinucleate) cells, consistent with pigmented villonodular synovitis.

Because of the patient’s persistent pain and limited range of motion for nearly 20 months despite activity modification, home exercises, and NSAIDs, along with the abnormal MRI findings, surgery was recommended. The patient elected to proceed and was taken to the operating room for arthroscopic synovectomy, debridement, and subpectoral biceps tenodesis. The patient was placed in the beach-chair position with an articulated arm holder under general anesthesia and interscalene block. Routine arthroscopic portals were placed including posterior, anterior, and anterolateral portals. Diagnostic arthroscopy with a 30° arthroscope revealed extensive, global proliferative synovitis throughout the entire glenohumeral joint including the axillary pouch, with numerous yellow-brown to rust-colored papillary projections (Figure 2). A 70° arthroscope was also used to enable visualization of the periphery of the joint, and multiple synovial biopsies were taken. Exuberant synovitis was seen immediately anterior and posterior to the subscapularis tendon. The rotator cuff and glenohumeral ligaments were intact. There were no chondral lesions of the humeral head or glenoid. The long head of the biceps tendon (LHB) appeared normal, though the biceps anchor and superior labrum were hypermobile, consistent with a type 2 unstable SLAP (superior labral tear from anterior to posterior) tear. Total arthroscopic synovectomy was performed with a 3.5-mm full-radius shaver (Figure 3). Given the type 2 SLAP tear and MRI showing abnormal tissue distal to the bicipital groove, it was felt that an arthroscopic biceps tenotomy with subpectoral tenodesis and open synovectomy was indicated. The LHB tendon was arthroscopically tenotomized at its superior labral origin. A 2-cm longitudinal incision was made just adjacent to the axillary fold over the anteromedial aspect of the proximal humerus, with its superior extent at the inferior border of the palpable pectoralis major tendon. The LHB tendon sheath was longitudinally incised, revealing extensive synovial proliferation including a large, hypertrophic synovial lesion extending proximally beneath the pectoralis major tendon. The LHB tendon was retrieved and tenodesed within the bicipital groove with a 2.8-mm all-suture anchor, allowing for complete removal of diseased synovial tissue within the bicipital groove. Postoperatively, the patient was maintained in a sling for three weeks with initiation of pendulum exercises and passive range of motion at the elbow, wrist, and hand. She was instructed not to lift anything heavier than utensils for six weeks to protect the biceps tenodesis. Surgical histopathology demonstrated multiple pink-tan to yellow-brown fragments of tenosynovial giant cell tumor, diffuse type, otherwise known as PVNS (Figure 4). The patient provided consent for this case to be submitted for publication.

## Discussion

PVNS of the shoulder is extremely rare and, as such, may evade clinical suspicion and delay diagnosis and treatment. The management of diffuse-type shoulder PVNS may include open or arthroscopic synovectomy and debridement [7,8,10,12,18] versus arthroplasty [16,19,20], with the possible adjuvant use of radiation therapy [8], depending on the severity and extent of disease, presence of extra-articular disease, and surgeon experience.

Meticulous surgical technique will increase the likelihood of successful arthroscopic treatment of PVNS within the shoulder (Table 1). As compared with an open approach, we feel arthroscopic synovectomy provides better visualization and access to all compartments of the shoulder and reduces the likelihood of residual disease, along with reducing surgical morbidity and postoperative recovery for the patient. The surgeon must thoughtfully place portals, including accessory portals as needed, with the intent of alternating working and viewing portals for optimal visualization and access. The use of a 70° arthroscope in conjunction with a 30° arthroscope enhances access to the joint periphery. It is also helpful to have straight and curved arthroscopic shavers available, along with graspers and tissue biters, utilizing the different portals to assure complete removal of the diseased synovium and any loose bodies encountered. A high index of suspicion should be maintained for comorbid rotator cuff tears and chondral lesions, and these should be concurrently addressed. A thorough synovectomy should be performed in a systematic fashion to lower recurrence risk, taking care not to leave any remnant disease. Dissection should begin by establishing the tissue plane between the normal synovial lining and diseased synovium, ensuring adequate resection depth by removing a rim of underlying healthy synovium. Although the proliferative synovium in PVNS is sometimes described as hypervascular, we did not find hemostasis to be a challenge with addition of epinephrine to the arthroscopic irrigation fluid and adjustment of arthroscopic pump pressure as needed. Radiofrequency devices can be used for hemostasis and tissue ablation but should be used judiciously to avoid excessive heat generation and thermal chondral insult.

**Table 1:** Pearls and pitfalls in the arthroscopic treatment of PVNS involving the shoulder. PVNS, pigmented villonodular synovitis

Pearls and pitfalls
Ensure that 30° and 70° arthroscopes are available
Achieve accurate portal placement with alternate working and viewing portals for optimal visualization and access
Ensure that multiple types of arthroscopic shavers (straight and curved, flexible and rigid) are available and cycle these between portals
Ensure graspers and/or biters are available as synovial tissue in PVNS may be more resistant to standard shavers and may require more robust instruments
Be prepared to identify and remove loose bodies not necessarily evident on MRI
Be prepared to identify and address comorbid rotator cuff tears and/or chondral lesions
Perform a thorough synovectomy in a systematic fashion to lower recurrence risk
Begin dissection by establishing tissue plane between normal synovial lining and diseased synovium
Exercise judicious use of radiofrequency ablation to avoid excessive heat generation and thermal chondral insult

## Conclusions

In summary, clinicians should be cognizant of PVNS in their differential diagnosis for insidious, permeative soft tissue lesions of the shoulder that present with atraumatic pain, recurrent effusion, and painful shoulder motion. Accurate and timely diagnosis are important to ameliorate pain and stiffness and intervene prior to irreversible joint destruction. Although open synovectomy with arthroplasty may be considered for more severe cases with advanced, bipolar chondral damage, arthroscopic synovectomy and debridement with LHB tenodesis is a viable treatment option for patients with diffuse-type shoulder PVNS. Meticulous attention to surgical technique is needed to ensure complete synovectomy and minimize the risk of local recurrence. Further study is needed to characterize the natural history and prognosis of this disease following surgical treatment, determine best surveillance practices to detect subclinical recurrence at follow-up, and define a treatment algorithm for recalcitrant cases.
